# D-cycloserine effects on COPD and depression in a murine experimental model

**DOI:** 10.3389/fphar.2025.1554337

**Published:** 2025-05-30

**Authors:** Uriel Heresco-Levy, Jacob Haviv, Yehezkel G. Caine, Jimmy Bao, Tai-Yu Huang, Chien-Chang Shen, Naama R. Bogot

**Affiliations:** ^1^ Herzog Medical Center, Jerusalem, Israel; ^2^ Psychiatry Department, Hadassah Medical School, Hebrew University, Jerusalem, Israel; ^3^ Pharmacology Discovery Services Taiwan, Ltd., New Taipei City, Taiwan; ^4^ Eurofins Discovery Services North America, LLC, St. Charles, MO, United States; ^5^ Cardiothoracic Radiology Unit, Shaare Zedek Medical Center, Jerusalem, Israel

**Keywords:** COPD, depression, inflammation, NMDAR modulation, D-cycloserine, lung, brain

## Abstract

**Background and Purpose:**

Both chronic obstructive pulmonary disease (COPD) and depression are associated with chronic inflammation and their comorbidity represents a critical unmet treatment need. N-methyl-D-aspartate glutamatergic receptors (NMDAR) are well characterized in CNS and widely expressed in lung tissue and inflammation-related cells. Pathologic NMDAR activation, leading to proinflammatory signaling, reactive oxidative stress and tissue damage plays a crucial role in chronic lung injury and depression. D-cycloserine (DCS), an antitubercular antibiotic, acts also as a NMDAR functional antagonist and has antidepressant and anti-inflammatory effects. We hypothesize that NMDAR downregulation represents a unified molecular target for the treatment of COPD–depression comorbidity. This study assessed whether DCS can ameliorate lung injury and depression–like behavior in the porcine pancreatic elastase (PPE)/*E.coli* lipopolysaccharide (LPS) murine COPD model.

**Material and Methods:**

Male BALB/c mice 7–8 weeks old received PPE intratracheally (IT) (1.2 U/20 µL/mouse) on days 0, 7, 14 and 21 and LPS (7 µg/20 µL/mouse) on days 4, 11, 18 and 25 (Groups 2–5). Sham control mice (Group 1) received same volume of saline IT in the same schedule as PPE and LPS. Vehicle (saline) or DCS 100 or 200 mg/kg were administered intraperitoneally once daily from day 28 to day 34 (Groups 2–4). An additional group (Group 5) received DCS 100 mg/kg once weekly (days 7, 14 and 21) and once daily from day 28 to day 34. On day 35 mice underwent the forced swim test (FST) and lungs were harvested for histopathological analyses.

**Results:**

Inflammatory cell infiltration, focal emphysema, measured by the mean linear intercept (MLI), and FST immobility duration, a rodent proxy for depression, were all increased (p < 0.05) in the vehicle group. In comparison with the vehicle group, immobility duration was reduced (p < 0.05) in both DCS 100 mg/kg groups. Moreover, the severity grading of lung inflammation and MLI were reduced (p < 0.05) in the DCS 100 mg/kg × 10 group and in all DCS-treated groups, respectively.

**Conclusion:**

Our findings suggest beneficial DCS effects and warrant further DCS investigation as an innovative treatment for COPD-depression comorbidity.

## Introduction

Chronic obstructive pulmonary disease (COPD) is a multicomponent slowly-developing disorder with a 7.4%–12.6% global prevalence that represents one of the main causes of death (Varmaghani et al., 2019; Al Wachami et al., 2024). Main COPD characteristics include pulmonary inflammation, alveolar destruction and airway obstruction (Vogelmeier et al., 2017). One cluster of COPD-related comorbidities involves the brain and mental functions. Depression is common in patients with COPD (Yohannes, 2018; Al Wachami et al., 2024). In two meta-analyses, the prevalence of depression in COPD was found to be 24.6% and 27.1% while in non-COPD subjects the prevalence was 11.7% and 10.0% (Matte et al., 2016; Zhang et al., 2011). In a longitudinal study of 35,000 patients with COPD and with a follow-up of 10 years, the incidence of depression was 16.2 cases per 1,000 person-years in the COPD group compared with 9.4 cases per 1,000 person-years in the non-COPD control group. In addition, those with severe COPD were twice as likely to develop depression compared with patient with mild COPD (Schneider et al., 2010; Atlantis et al., 2013). Overall, one in four individuals with COPD-depression comorbidity experience an unfavorable illness course (Yohannes et al., 2010; Atlantis et al., 2013; Vikjord et al., 2020) Decline in functional parameters and quality of life as well as increased number of hospitalizations and mortality risk are all characteristics at this comorbidity (Yohannes et al., 2016; Lou et al., 2014).

The drugs commonly used to treat COPD e.g., glucocorticoids and bronchodilators, have significant side effects and do not alleviate depression. Add-on selective serotonin reuptake inhibitors and serotonin- norepinephrine reuptake inhibitors are employed for this indication. However, their efficacy and safety in COPD patients remains controversial (Yohannes, 2018; Vozoris et al., 2018). Proposed mechanisms by which serotonergic antidepressants may cause harm in COPD include induction of sleepiness leading to decreased oxygen and increased carbon dioxide levels, lowering of infection threshold and reduction of apoptotic cells clearance (Yohannes, 2018; Martínez-Gestoso et al., 2022). Thus, efficient treatment of COPD-depression comorbidity represents a critical unmet need.

N-methyl-D-aspartate receptors (NMDAR) are complex cation channel glutamate (Glu) receptors widely expressed and well characterized in the central nervous system (CNS) that are well-acknowledged to play a crucial role in brain physiological functions, excitotoxicity and neuropsychiatric disorders. Functional NMDAR comprise two obligatory NR1 subunits, binding the co-agonists glycine and D-serine (DSR) at the glycine modulatory site (GMS), and two Glu-binding NR2 (2A-D) subunits, or a combination of NR2 and NR3 (3A-B) subunits. Different subunit combinations endow NMDAR with distinct physiological and pharmacological properties (Stroebel and Paoletti, 2021; Geoffroy et al., 2022). Moreover, accumulating evidence suggests that Glu also acts as a signaling molecule outside the CNS, with an emerging role as an immune modulator. In line with this concept, functional glutamatergic signaling and NMDAR expression have been found extensively in non-neuronal tissues, including lung, kidneys, heart, pancreatic β cells, lymphocytes and inflammation-related cells (Affaticati et al., 2011; Boldyrev et al., 2012; Bozic and Valdivielso, 2015; Ma et al., 2020). Physiological levels of NMDAR activation are of paramount importance and NMDAR hyperactivation plays a significant role in inflammation and depression as well as in COPD. Under pathological conditions, extracellular Glu concentrations are increased by abnormal release and/or clearance. The resulting NMDAR over-activation triggers rapid Ca^2+^ influx in the cell that may lead to activation of proinflammatory signaling pathways and reactive oxidative stress (ROS), ultimately resulting in cell damage and organ dysfunction (Trynelis et al., 2010; Gonzalez et al., 2015; Bozic and Valdivielso, 2015; Ma et al., 2020).

Research supports the concept that inflammation and depression are associated and neuroinflammation affects up to 27% of patients with major depressive disorder (MDD) (Hassamal, 2023). Low-grade inflammation represents, partly via NMDAR activation, a potential pathophysiological mechanism in depression. NMDARs are expressed on immune cells that may release GLU endogenously (Boldyrev et al., 2012; Ma et al., 2020) and C-reactive protein (CRP), various cytokines and TNF-α are increased in patients with depression. Some studies point to a role for increased inflammation specifically in patients with treatment-resistant depression. In MDD patients who attempt suicide, increased IL-1, IL-6 and CRP correlating with brain Glu levels were reported (Roman and Irwin, 2020; Suneson et al., 2021). Inflammation effects on glia initially lead to an increased release and “spillover” of Glu into the extrasynaptic space by decreasing the capacity of glial transporters to buffer and clear Glu. This Glu spillover in combination with Glu released by activated or primed glial and immune cells can activate extrasynaptic NMDAR and lead to atrophy and regression of dendritic spines and processes and loss of synaptic integrity, ultimately resulting in neuronal loss (Krystal et al., 2013; Haroon et al., 2017). Currently, significant efforts are invested in the development of glutamatergic antidepressants. Intranasal spray of esketamine (Spravato), the active enantiomer of ketamine, a non-competitive NMDAR channel blocker and dissociative anesthetic is presently the first glutamatergic antidepressant approved for use, under precautionary conditions for refractory MDD and suicidality.

While the expression and function of NMDAR in CNS have been studied extensively, their presence in non-neuronal tissues, including lung, is largely unexplored. Nevertheless, Glu signaling and NMDAR are expressed in various lung regions and cell types including alveolar type II cells, trachea and airways (Dickman et al., 2004; Affaticati et al., 2011; Anaparti et al., 2015). The NMDAR GluN1/2C subtype was found to be expressed in peripheral and middle-lobe lung samples, the GluN1/2D subunit was predominantly expressed in the peripheral, gas-exchange zone of the lungs and in alveolar macrophages; this expression was upregulated in lungs treated with N-methyl-D-aspartate (Dickman et al., 2004). NMDAR involvement has been reported in airway perfusion, contractile responses, tracheal muscle tone and fibroblast proliferation and differentiation (Anaparti et al., 2015; Foutz et al., 1988; Dong et al., 2021). Moreover, functional NMDAR are expressed on immune cells (i.e., mononuclear leukocytes, neutrophils, dendritic cells, macrophage and platelets) that may release GLU endogenously (Affaticati et al., 2011; Boldyrev et al., 2012).

*In vitro* and *in vivo* observations strongly indicate an involvement of Glu toxicity and NMDAR hyperactivation in lung injury and COPD. NMDAR activation by Glu released from damaged lung and immune competent cells contributes to oxidant lung injury, resulting in increased ROS and caspase-3 activation (Said et al., 2000; Ma et al., 2020). Administration of the NMDAR noncompetitive channel antagonist MK-801 protects against oxidative stress in lipopolysaccharide–induced acute lung injury in rat (da Cunha et al., 2010). In COPD mouse model and cigarette smoke-treated (CS-treated) Raw 264.7 cells, NR1 was upregulated and resulting pulmonary inflammatory responses and increased Glu levels were ameliorated by the noncompetitive NMDAR antagonist memantine (Cheng et al., 2019). In several chronic lung injury animal models and CS-treated mouse macrophage cells, cysteine/glutamate transport was upregulated, prompting the release of endogenous Glu. Furthermore, the over-activation of NMDAR in macrophages also prompted pro-inflammatory cytokine secretion via Ca^2+^-mediated phosphorylation of extracellular regulated protein kinases 1/2 (ERK 1/2). In this way, abnormally activated NMDARs aggravate inflammatory responses, regulate the functions of fibroblasts and bone marrow mesenchymal stem cells (BM-MSC) and contribute to lung disease (rev in Ma et al. (2020)). Moreover, preliminary data implicate NMDAR hyperstimulation in the pathogenesis of additional chronic lung disorders, i.e., asthma, pulmonary fibrosis and pulmonary arterial hypertension (Anaparti et al., 2015; Li et al., 2018; Dumas et al., 2018).

In view of these convergent lines of evidence, we hypothesized that NMDAR may represent a unified molecular target for COPD-depression comorbidity and that pharmacological NMDAR downregulation may result in alleviation of respiratory and depression symptomatology. We further postulate that treatment with D-cycloserine (DCS, Seromycin, Cycloserine), a broad-spectrum antibiotic used in tuberculosis (TB), may achieve these dual-hit objectives (Heresco-Levy et al., 2024).

DCS, a structural analogue of D-alanine, is a *streptomyces*-isolated broad spectrum antibiotic approved for TB treatment, used since the 1950s, usually at 500–1000 g/day regimens, in millions of individuals. DCS blocks bacterial growth by inhibiting alanine racemase and D-alanine ligase. In the 1980s it became evident that in addition to its bacteriostatic mechanisms, DCS is also a selective NMDAR NR1 partial agonist that appears to act via allosteric modulation of GMS. *In vivo* DCS acts like an agonist at low doses but has NMDAR antagonistic features with high doses (Schade and Paulus, 2016).

Since the discovery of its NMDAR effects, DCS has become the focus of intense research in neuropsychiatric disorders (Heresco-Levy and Javitt, 2008; Durrant and Heresco-Levy, 2013). In a variety of brain injury animal models, it was shown that DCS can reverse synaptic plasticity alterations via improved long-term potentiation (LTP), restore brain derived neurotropic factor (BDNF) levels and induced higher dendritic spine density (Yaka et al., 2007; Na et al., 2017; Wu et al., 2018).

DCS antidepressant effects in tuberculosis patients were noted already in the 1950s (Crane, 1959). More recently, high-dose add-on DCS was shown to safely relieve treatment-resistant depression (TRD), with therapeutic effects evident 2–4 weeks after treatment initiation and with no associated psychotic or dissociative side effects as registered with direct NMDAR channel blockers (Heresco-Levy et al., 2013). A potential role of DCS in the maintenance of the antidepressant effect of a single dose of ketamine was suggested in patients with bipolar depression (Kantrowitz et al., 2015). A therapeutic benefit of DCS maintenance treatment has been demonstrated for TRD patients who respond to ketamine infusion but have a residual suicidal risk (Chen et al., 2019). Moreover, recently specific DCS anti-inflammatory mechanisms were reported. In LPS-induced RAW 264.6 macrophage cell lines, DCS inhibited nitric oxide (NO) production in a concentration-dependent manner and supressed the expression of pro-inflammatory cytokines such as interleukin IL-1β and IL-6 (Kang and Hyun, 2020).

In view of these accumulating data and in order to explore our hypotheses, we assessed in the present study the effects of various dose regimens of DCS in the elastase-induced murine model of COPD and LPS-induced pulmonary inflammation (Sohn et al., 2013; Pelgrim et al., 2022). The forced swim test (FST) was performed in order to evaluate eventual DCS antidepressant effects and lung post-mortem analyses were executed to evaluate DCS administration effects upon pulmonary inflammation and emphysema.

## Materials and methods

### Animals and experimental design

The experiments were performed by Pharmacology Discovery Services, Taiwan, partner laboratory of Eurofins Pharma Discovery Services. Male BALB/c mice, 7–8 week-old, were provided by BioLASCO Taiwan (under Charles River Laboratory Licensee). Space allocation for individual animals was 30 × 19 × 13 cm^3^. All animals were maintained in an environment under well-controlled temperature (20°C–24°C) and humidity (30%–70%) with 12 h light/dark cycles. All experimental procedures were performed in the light phase. Mice had *ad libitum* access to standard lab diet [MFG (Oriental Yeast Co., Ltd., Japan)] and autoclaved tap water. All aspects of this work including housing, experimentation, and animal disposal were performed in accordance with the “Guide for the Care and Use of Laboratory Animals” (National Academies Press, 2011) in our AAALAC-accredited laboratory animal facility. In addition, the animal study was reviewed and approved by the Institutional Animal Care and Use Committee (IACUC) (IN044-01302023-46122) at Pharmacology Discovery Services Taiwan, Ltd.

**FIGURE 1:**
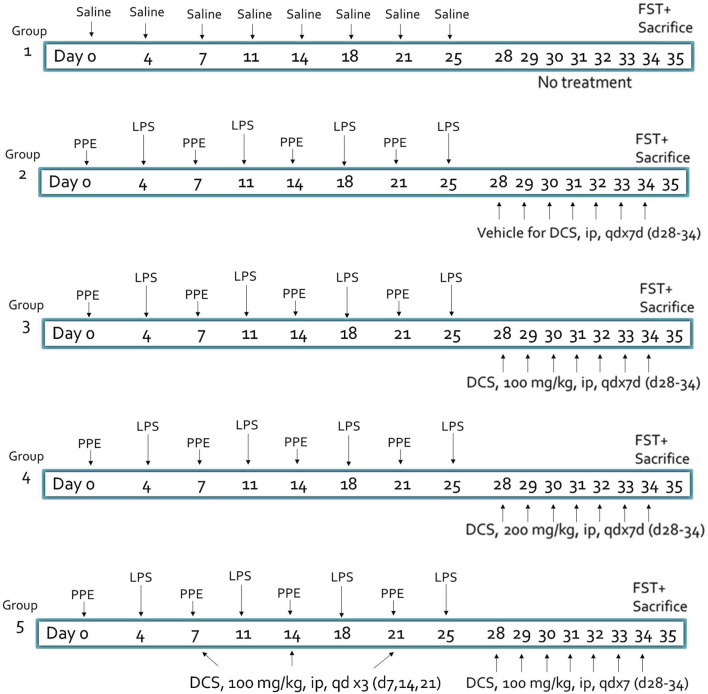
Experimental design and treatment groups. Animal: male BALB/c mice, 7–8 weeks old, N = 8 per group. PPE, porcine pancreatic elastase (1.2 U/20 µL/mouse), LPS, lipopolysaccharide (7 µg/20 µL/mouse) – administered intratracheally. DCS, D-cycloserine, adminstered intraperitonealy. FST, forced swim test.

Animals were randomly divided into five study groups (Figure 1): negative control (Sham) (Group 1), vehicle control (saline) (Group2) and three DCS treatment groups (Group 3–5). All animals were anesthetized with isoflurane and except those in Group 1, received intratracheal instillation of porcine pancreatic elastase (PPE) at a dose of 1.2 U/20 µL/mouse on Days 0, 7, 14 and 21 and *E. Coli* lipopolysaccharide (LPS) at a dose of 7 µg/20 µL/mouse on Days 4, 11, 18 and 25. Sham control mice (Group 1) received the same volume of saline intratracheally in the same way and same schedule as PPE and LPS, i.e., on Days 0, 4, 7, 11, 14, 18, 21 and 25. Vehicle (saline) (Group 2) or test article, DCS at 100 mg/kg (Group 3) or 200 mg/kg (Group 4) were administered by intraperitoneal injection (IP) once daily (QD) from Day 28 to Day 34. Group 5 received DCS once weekly (Days 7, 14, and 21) followed by once daily (QD) from Day 28 to Day 34. On Day 35 mice underwent the FST, following which lungs were harvested for histopathological analyses.

Mice body weights were recorded three times per week during the study period.

### Materials

Elastase, Porcine Pancreas (PPE) (324682) (Sigma, United States), Isoflurane (Panion and BE biotech, Taiwan), Lipopolysaccharide (LPS, 055:B5) (Sigma, United States), 0.9% NaCl (Sintong Chemical Industry Co., Ltd. Taiwan), Pentobarbital sodium (Health-Tech Pharmaceutical Co., Ltd., Taiwan), and water for injection (WFI) (Tai-Yu, Taiwan).

The test article, DCS (Sigma, United States), was purchased by Pharmacology Discovery Services Taiwan, Ltd. The DCS formulations used are summarized in Table 1.

**TABLE 1:** D-cycloserine study formulations.

Test compound	Vehicle	Solubility^a^	Color	Light protection^b^	Temperature^c^	Concentration (mg/mL)
D-cycloserine	0.9% NaCl	S	Colorless	N	4°C	10
D-cycloserine	0.9% NaCl	S	Colorless	N	4°C	20

^a^ This is based upon visual observation. S, soluble; SS, slight soluble; I, insoluble (suspension or precipitation).

^b^ Y, formula is kept in tube or vial with brown color or covered with aluminum foil. N, no protection from light.

^c^ RT, prepared fresh and stored between 20°C and 25°C. 4°C: prepared fresh and stored in the refrigerator or kept on ice.

### Forced swim test

The forced swim test (FST) was performed following DCS administration, in order to detect a signal antidepressant effect of the test compound in mice with recently -induced emphysematous and inflammatory injury. FST is a rodent depression model which measures the amount of time a mouse swims in a restricted space before “giving up” and becoming immobile. Reduction of immobility time in this assay predicts antidepressant effects across a range of antidepressant compound types (Cryan et al., 2005).

Animals were placed in a 1-L volume glass beaker (height: 14.5 cm, diameter: 11 cm) containing 10 cm height water at room temperature (22°C–24°C) for 6 min. The first 2 min were considered a time for the animals to explore and acclimate to the environment. The duration of animal immobility within the last 4 of the 6 min was then recorded. As a second readout the fourth and fifth minutes (time 180–300 s) was evaluated. A mouse was considered immobile when it ceased struggling and remained floating in the water making only those movements necessary to keep its head above water. Beside immobility, also the parameters swimming and struggling were evaluated.

### Histopathology

Following necropsy, at day 35, lung samples were harvested and preserved in 10% neutral buffered formalin (NBF). A total of four lung lobes (R1: Right cranial lobe, R2: Right middle lobe, R3: Right caudal lobe and A4: Accessory lobe) were filled in with formalin through a tracheal cannula and processed for histopathological analysis. The tissue was then dehydrated and embedded in paraffin and the lung tissues were sectioned at 4–6 µm. Formalin-fixed, paraffin-embedded slides were immersed thrice in xylene for 3 min to dewax. These were then moved through graded ethanol concentrations (100%, 90%, 80%, and 70%) for 3 min each, followed by deionized water for 3 min and 30 s. The slides were stained with hematoxylin, followed by tap water for 10 min and then stained with eosin. Next, slides were rapidly dehydrated through graded ethanol concentrations (80%, 90%, 95%, and 100%) for 5 or 15 s each, before clearing in xylene and mounting. Finally, samples were examined through an optical microscope (Leica DM2700M, United States).

### Quantitative recording of histopathological lesions of the lung (inflammation)

The severity of inflammation cell infiltration was used to evaluate the pulmonary inflammation score using previously employed criteria (Shackelford et al., 2002). Whole lung was used including parenchyma and airways. The pathological veterinarian conducted blind test interpretation analysis without knowing which test article was used in each test group using semi-quantitative scoring criteria to assess histopathology, as previously described (Shackelford et al., 2002). The severity grading system for all microscopic lesions (H&E stain) ranged from 0–5 as follows: Grade 0 = Not present, Grade 1 = Minimal (<1%), Grade 2 = Slight (1%–25%), Grade 3 = Moderate (26%–50%), Grade 4 = Moderately severe/High (51%–75%), Grade 5 = Severe/High (76%–100%).

### Assessment of emphysema

To assess air space enlargement, as a measure of emphysema, the mean linear intercept (MLI) was quantified by one observer in a blinded fashion by superimposing a line grid with a line on the images of lung enlargement sections at a magnification of 100X as described previously (Knudsen et al., 2010). To calculate the MLI, the number of intersections between the lines of the grid and the alveolar walls was quantified for each mouse in 5 non-overlapping fields. Air space enlargement was quantified by the mean linear intercept (MLI) calculations in randomly selected fields of the lung tissue sections. MLI was obtained by dividing the total length of a line drawn across the lung section by the total number of intercepts encountered. The point-counting technique determined the mean linear intercept (×100 magnification) in the pulmonary tissue across 5 random, non-overlapping microscopic fields.

### Statistical analysis

All values were expressed as mean ± SEM or mean ± SD. The unpaired Student’s t-test compared vehicle control (Group 2) vs. sham (Group 1). One-way ANOVA followed by Dunnett’s test were used to determine possible significant (*p* < 0.05) differences between the vehicle control (Group 2) vs. treated groups (Groups 3–5), All analyses were performed using GraphPad Prism 8 or IBM SPSS Statistics 22.0.

## Results

**FIGURE 2:**
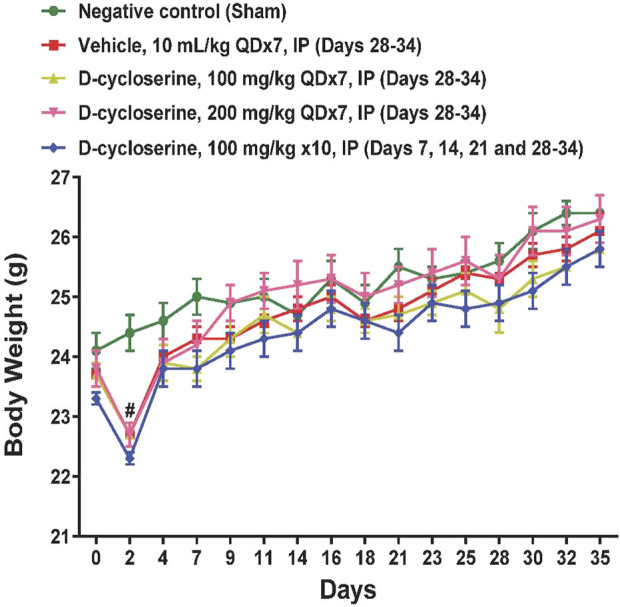
Body weights during the study. Values represent mean ± SEM. #*p* < 0.05, treated vs Sham, two-way ANOVA followed by Bonferroni test.

DCS was well-tolerated at all tested dose levels and no overt toxicities were observed during the study. The body weight in sham control mice increased gradually during the whole study. Only on Day 2 the body weights were significantly (p < 0.05) reduced in the COPD induction groups (Figure 2).

### Lung inflammation

**FIGURE 3:**
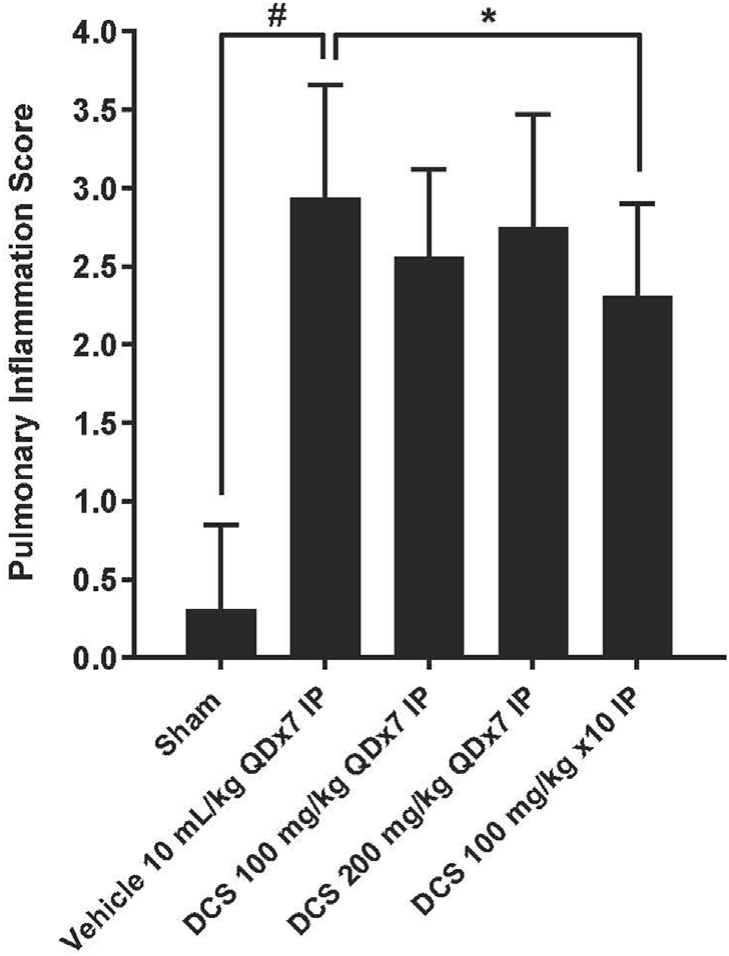
Pulmonary inflammation assessment in lung histological sections. Values represent mean ± SD. #*p* < 0.05, Sham (Group1) vs Vehicle control (Group 2); unpaired Student’s t-test. *p < 0.05, Treated (Group 3- Group 5) vs. Vehicle control (Group 2); one-way ANOVA followed by Dunnett’s test.

Focal to multifocal, slight to moderate inflammatory cell infiltrations were found in all groups treated with PPE and LPS. The severity of inflammation in the vehicle group (Group 2) (2.94 ± 0.72) was significantly increased compared to the sham control group (Group 1) (0.31 ± 0.54). The severity of inflammation in groups 3–5, which received PPE and LPS, but were subsequently treated with DCS, was reduced in comparison with the vehicle control group. In group 5, which received DCS 100 mg/kg/day for three times during PPE/LPS exposure, as well as 100 mg/kg for 7 days following PPE/LPS administration period, the severity of lung inflammation (2.31 ± 0.59) was significantly (p < 0.05) reduced in comparison to the vehicle control (Figure 3).

### Emphysema

**FIGURE 4:**
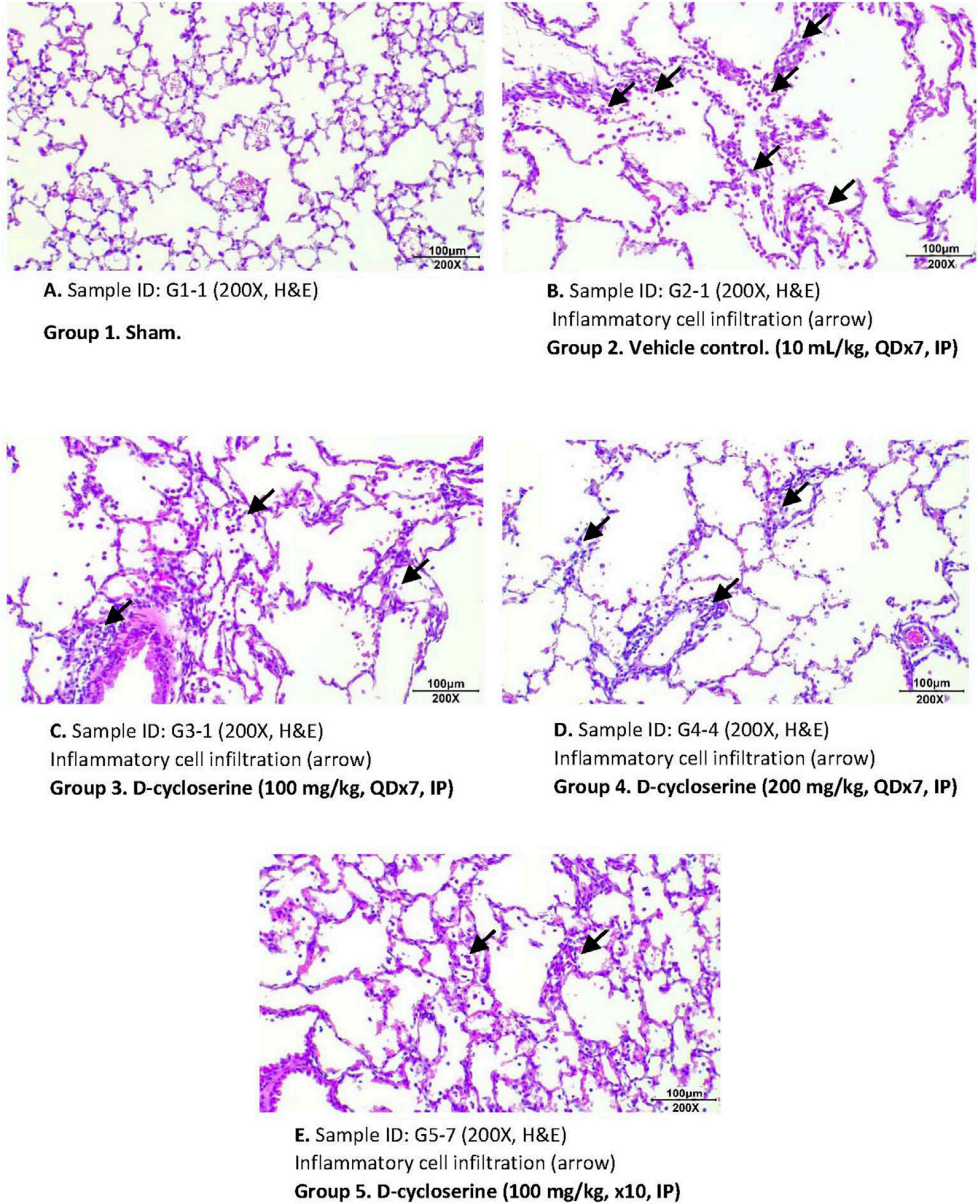
Representative photomicrographs of H&E - stained lung sections for each study group. **(A)** Group 1. Sham. Sample ID: G1-I (200X, (H&E). **(B)** Group 2. Vehicle control. (10 mL/kg, QDx7, IP). Sample ID: G2-I (200X, H&E). **(C)** Group 3. D-cycloserine (100 mg/kg, QDx7, IP). Sample ID: G3-I (200X, H&E). **(D)** Group 4. D-cycloserine (200 mg/kg, QDx7, IP). Sample ID: G4-4 (200X, H&E). **(E)** Group 5. D-cycloserine (100 mg/kg, x10, IP). Sample ID: G5-7 (200X, H&E).

**FIGURE 5:**
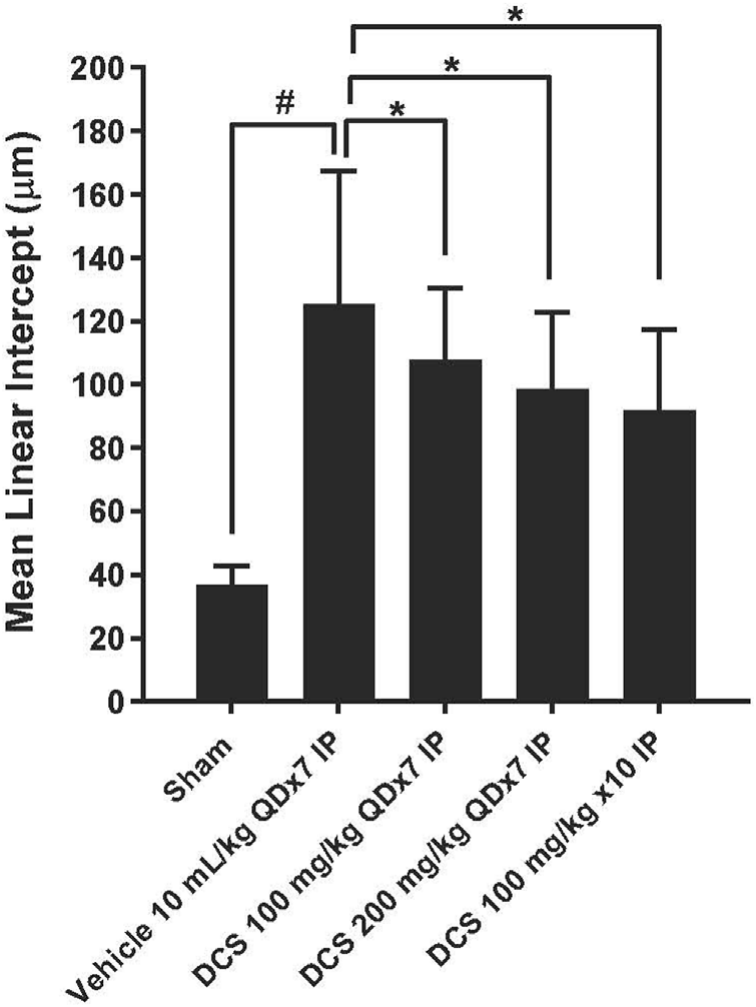
Emphysema assessment in lung histological sections. Values represent mean ± SD. *#p* < 0.05, Sham (Group 1) vs. Vehicle control (Group 2); unpaired Student’s t-test. *p < 0.05, Treated (Group 3 - Group 5) vs. Vehicle control (Group 2); one-way ANOVA followed by Dunnett’s test.

To assess emphysema, MLI was measured in histological sections of lung tissue. Photomicrographs of these sections show enlarged air spaces in the PPE/LPS-exposed animals (Figure 4). In the vehicle group (Group 2), MLI was significantly augmented as compared to the sham group (Group 1) (125.38 ± 41.86 vs. 36.85 ± 6.04 µm). In all regimen/dose DCS-treated groups (Groups 3–5) a significant (p < 0.05) improvement in MLI was registered when compared to the vehicle control group. The greatest MLI reduction (92.02 ± 25.45 µm) was registered in Group 5 which received DCS during as well as post-PPE/LPS administration (Figure 5).

### Forced swim test

The forced swim test was performed on Day 35, following PPE/LPS administration and DCS treatment. In this test of depressive-like behavior, mice in the negative control (sham) group processed an average immobility time of 124.4 ± 16.4 s during the last 4-min recording period (120–360 s), accompanied by duration of immobility, struggling (climbing) and swimming of 63.0 ± 8.3, 2.9 ± 1.2 and 54.1 ± 8.1 s, respectively, in the intervals of 3–5 min. Moreover, the vehicle control processed an average immobility time of 180.5 ± 8.3 s during the last 4-min recording period (120–360 s), accompanied by duration of immobility, struggling (climbing) and swimming of 84.8 ± 6.8, 1.4 ± 0.8 and 33.9 ± 6.9 s, respectively, in the intervals of 3–5 min.

**FIGURE 6:**
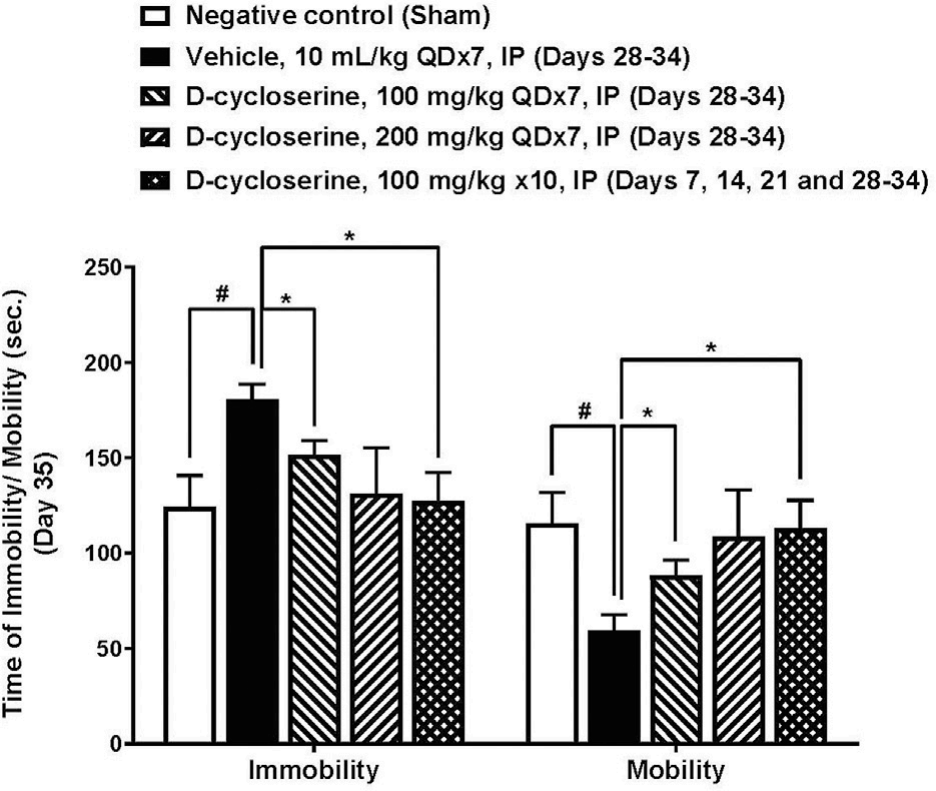
Forced swim test parameters at day 35. Values represent mean ± SEM. #*p* < 0.05, Sham (Group 1) vs. Vehicle control (Group 2); unpaired Student’s t-test. *p < 0.05, Treated (Group 3–5) vs. Vehicle control (Group 2); one way ANOVA followed by Dunnett’s test.

The PPE/LPS-induced injury in the vehicle group was associated with significantly increased total immobility time or significantly reduced total mobility time compared to the sham group, (p < 0.05, Figure 6). Duration of immobility was reduced and duration of mobility increased in all DCS treated groups. Moreover, DCS given at 100 mg/kg, QD x7 (Days 28–34) and at 100 mg/kg, × 10 (Days 7, 14, 21, 28–34) induced significant (p < 0.05) reductions in the total duration of immobility (151.5 ± 7.9 and 127.3 ± 15.0 s, respectively) and improvement on the total duration of mobility as compared to the vehicle group (Figure 6).

## Discussion

Systemic inflammation and NMDAR overactivation are implicated in COPD pathophysiology (Pelgrim et al., 2019; Ma et al., 2020; Heresco-Levy et al., 2024). A number of studies examined whether downregulation of brain NMDAR may be beneficial in COPD comorbidities. Inhibition of NMDAR by lovastatin, a classic cholesterol lowering drug, ameliorated cognitive deficits in a chronic intermittent hypoxia hypercapnia animal model of COPD (Huo et al., 2014). Since DCS is a brain active drug known to promote neuronal plasticity and expectation–related learning (Monahan et al., 1989; Reinecke et al., 2020), one study examined whether add-on DCS may augment the effects of pulmonary rehabilitation in COPD patients. Although overall no significant DCS effects were found on study outcomes it was observed that DCS was independently associated with breathlessness improvement (Finnegan et al., 2023a; Finnegan et al., 023b). The present study represents, to the best of our knowledge, the first investigation of DCS effects in a murine model of COPD. Overall, our findings suggest that DCS may have favorable lung and behavioral effects in the context of COPD-depression comorbidity.

With respect to the pulmonary and behavioral phenotypes, the employed model includes core characteristics of COPD pathology (Sohn et al., 2013; Pelgrim et al., 2022). Elastase exposure resulted in our study in significant emphysema as reflected by an increased MLI value. Moreover, the recurrent LPS exposure resulted in significant inflammatory cell infiltration and increased inflammation severity scores. In regard to extra-pulmonary manifestations, the administered PPE/LPS regimen resulted in increased immobility of the mice in FST-an established animal proxy of depressive behavior (Cryan et al., 2005). Our findings suggest, in accordance with the study hypotheses, that DCS may broadly attenuate these pathological manifestations: MLI magnitude was reduced in all DCS- treated groups, the severity of lung inflammation was reduced in the DCS 100 mg/kg × 10 group and FST immobility duration was reduced in the DCS 100 mg/kg × 7 as well as 100 mg/kg × 10 – treated groups.

Three DCS regimens were employed in the present study: 100 mg/kg/day and 200 mg/kg/day for 1 week following the 25 days PPE/LPS administration period, while an additional group (Group 5) received the same 100 mg/kg/day regimen but in addition also received three identical DCS administrations during the 25 days PPE/LPS–induction period. This amplified DCS regimen resulted in significant attenuation of all assessed parameters, suggesting that earlier DCS administration during the development of the pathohistological injury may carry advantages. However, presently this interpretation is speculative and warrants further research.

An important issue in the context of our study hypotheses are the molecular mechanisms by which the observed DCS effects were obtained. An increase in ROS ultimately resulting in inflammatory responses, alveolar enlargement and lung apoptosis are established pathophysiological mechanisms in COPD, that are mimicked by elastase administration (Limjunyawong et al., 2015; Ma et al., 2020; Pelgrim et al., 2022). The effects observed on MLI in this study strongly suggest a direct protective DCS effect at lung level. The involvement of NMDAR in lung injury includes oxidative stress accumulation in addition to inflammation and dysfunction of specific lung tissues and cells (rev in Ma et al., 2020). Moreover, the protective effects of NMDAR blockade or antagonism in lung injury animal models achieved with the NMDAR channel antagonists MK-801 (da Cunha et al., 2010), memantine (Cheng et al., 2019) and DCS in the present study further suggest that NMDAR downregulation may have therapeutic benefits in COPD. Regarding, the depression-like behavioral manifestations observed in the used murine model, as measured by FST, they may have been contributed by a direct impact of PPE/LPS on brain, or the development of a generalized inflammatory response. In addition, it is likely that the development of COPD leading to lower lung capacity and oxygenation has impacted upon mice FST performance. Nevertheless, the DCS protective effect was found to be manifest also in terms of mice physical performance. On the other hand, depression affects mobility and appetite. Moreover, physical wellbeing and depression are intertwined and hard to separate by paradigms that have a motor component, e.g., FST, Tail- Suspension Test. Thus, our experiment can not conclusively elucidate to what extent DCS affected depression directly or via its lung-related effects.

The capacity for NMDAR antagonism and downregulation is a recognized characteristic of DCS, that acts as a partial agonist at NMDAR-GMS and has antagonistic features with high doses (Schade and Paulus, 2016). Since DCS is a weak partial agonist at N2A and N2B containing NMDAR, with greater agonist efficacy at N2C and N2D-containing receptors (Danysz and Parsons, 1998; Sheinin et al., 2001), it has been suggested that DCS may preferentially facilitate or inhibit NMDARs subtypes and high dose DCS may attenuate executive cognitive deficits associated with suicide risk (Chen et al., 2019). Moreover, one of DCS mechanisms of action evidenced in animal studies is the increase in BDNF levels (Yaka et al., 2007), which are known to negatively correlate with oxidative stress levels (De Sousa et al., 2022). Overall, NMDAR downregulation and antioxidant properties may represent some of the mechanisms explaining DCS effects.

DCS use has a number of advantages versus NMDAR channel antagonists. Memantine, a non-competitive, low-affinity voltage-dependent NMDAR antagonist, that was shown to attenuate bleomycin-induced acute lung injury (Cheng et al., 2019) is used in dementia but has little clinical utility in depression (Kadriu et al., 2020). Although associated in early reports with detrimental neuropsychiatric effects, no propensity for addiction, abuse, or frontal brain neurotoxicity, that have been reported with ketamine, have been associated with DCS (Hwang et al., 2013; Ding et al., 2016; Henter et al., 2018; Li et al., 2019). Moreover, DCS is the only antibiotic that has been used in humans for almost seven decades that has evaded resistance selection in bacterial populations (David, 1971; Evangelopoulos et al., 2019). Presently DCS belongs and presently belongs to core second line treatment group C listed by WHO guidelines for treatment of multi-drug and extensively-drug resistant-TB (Caminero et al., 2010, World Health Organization, 2018). Interestingly, in the context of COPD treatments armamentarium, antibiotics have been recently recognized to have anti-inflammatory effects beyond their antimicrobial activity. Immunomodulatory properties were reported with the macrolide erythromycin in diffuse panbronchiolitis (Kudoh et al., 1998) and with azithromycin in COPD (Huckle et al., 2018). Presently, there is a scarcity of data concerning anti-inflammatory properties of other types/classes of antibiotics and their potential role as short and/or long term therapeutic intervention in COPD.

The present study has several limitations, including the small mice samples, lack of FST administration pre-DCS treatment and the short period of time for which the DCS treatment regimens have been applied. Regarding the sample magnitude, we were guided by efforts to minimize the suffering and number of animals used. While looking for a signal for DCS effects on FST, we wanted to avoid a habituation effect on FST performance; undoubtedly, further research should include, pre and post treatment FST administration and prolonged DCS treatment periods.

## Conclusion

In this study the effects of DCS, a long-standing antibiotic drug with partial agonist properties at the NMDAR- GMS, were assessed in a PPE/LPS murine model of COPD.

Our findings suggest DCS anti-inflammatory, anti-emphysema, and antidepressant effects and warrant further DCS investigation as an innovative treatment for COPD-depression comorbidity.

## Future directions

Additional preclinical studies with DCS in established rodent models of COPD and depression are indicated. DCS mechanisms of action should be further elucidated by the measurement of: 1) inflammation markers and cytokine release (systemic/lung) and 2) Glu and NMDAR hyperactivation parameters levels. An important related topic would be to determine whether within the framework of depression and inflammation the lung can also be affected. Depression-related measurements should aim to discriminate to what extent DCS improves mood via CNS and/or lung-level effects.

Subsequent work should establish the optimal recommended DCS regimens and compare them with the effects of an established antidepressant. Ultimately, randomized controlled clinical trials are the key test for the proposed use of DCS. Safety concerns regarding antibiotic resistance will need to be addressed and monitored before DCS use in COPD and depression. Drug resistant infections represent a major medical problem of our time. Nevertheless, recent research indicates that DCS has been used for six decades without significant appearance and dissemination of antibiotic resistant strains, making it an ideal model compound to understand what drives resistance evasion (Evangelopoulos et al., 2019). Moreover, it was shown that the rate of spontaneous mutations conferring resistance to DCS (mutation rate) is ultra-low in *M. tuberculosis* (David, 1971; Evangelopoulos et al., 2019).

## Data Availability

The raw data supporting the conclusions of this article will be made available by the authors, without undue reservation.
